# A 73-Year-Old Female Diagnosed With Dermatofibrosarcoma Protuberans in the Primary Care Setting: A Case Report and Literature Review of Misdiagnosed Cases

**DOI:** 10.7759/cureus.62547

**Published:** 2024-06-17

**Authors:** Natalia C Guerra, Maan Faraj, Alaine Ainsley, Fatin Sahhar, William J Smith

**Affiliations:** 1 Dermatology, Michigan State University College of Osteopathic Medicine, Detroit, USA; 2 Family Medicine, Detroit Medical Center-Sinai Grace Hospital/Michigan State University, Detroit, USA; 3 Family Medicine, Detroit Medical Center/Wayne State University School of Medicine, Detroit, USA; 4 Pathology, Detroit Medical Center/Wayne State University School of Medicine, Detroit, USA

**Keywords:** biopsy, clinical features, proper diagnosis, non-specialist physicians, surgical management, misdiagnosis, dfsp, dermatofirbosarcoma protuberans

## Abstract

Dermatofibrosarcoma protuberans (DFSP) is a rare, slow-growing, malignant tumor in the dermis and subcutaneous fat diagnosed by pathological and immunohistochemical examinations. This case report provides the dermatological findings of a 73-year-old woman with DFSP who presented to a primary care clinic with a longstanding nodular lesion on her left upper thigh. Dermatological examination showed a solitary, skin-colored violaceous/hyperpigmented nodule on the superior anteromedial portion of the left thigh. A punch biopsy revealed spindle cell proliferation, and diffuse CD34 positivity, confirming the diagnosis of DFSP. A dermatology referral was placed for further management and complete surgical excision. Patient underwent wide local excision (WLE) and has no recurrence to date. Unfortunately, DFSP is commonly misdiagnosed before skin biopsy which delays treatment. This case is significant because DFSP is not often diagnosed accurately outside the dermatology specialty and serves as a reminder to practitioners to use biopsies during the diagnostic process of skin findings to prevent the delay in management.

## Introduction

Dermatofibrosarcoma protuberans (DFSP) is a rare slow-growing soft tissue sarcoma, a locally aggressive subcutaneous mesenchymal tumor derived from dermal fibroblasts [1]. The incidence of DFSP is similar in men and women and commonly presents between the third and fourth decades of life, although cases have been reported in all age groups [2,3]. DFSP is uncommon and accounts for 0.1% of all malignancies and less than 2% of all sarcomas [2,4]. Histologically, DFSPs are composed of spindle cell proliferation in a storiform or matted pattern that extends to adipose tissue with immunohistochemical (IHC) staining that is characteristically CD34 positive and factor XIIIa negative [1].

Classic DFSP initially presents as small, firm, painless dermal plaques with subcutaneous thickening, or atrophic, non-protuberant lesions. The lesions gradually progress into their protuberant stage appearing as reddish-blue plaques or protruding violaceous smooth nodules [4,5]. Pathogenesis involves supernumerary chromosomes and chromosome 17 and 22 translocations resulting in the sustained activation of the platelet-derived growth factor (PDGF) β-chain gene (PDGFB) protein tyrosine kinase which leads to DFSP cell growth [2,6]. 

The diagnosis of DFSP is often delayed as it clinically mimics non-malignant dermatological pathologies such as keloid, epidermal cyst, or dermatofibroma [7]. A dermoscopy is a valuable tool in tumor recognition and findings may prompt neoplasm diagnosis [5]. Although DFSP has a relatively good prognosis, it has a high propensity for recurrence and a rare potential for distant metastasis [2-4]. Standard treatment for DFSP involves complete surgical resection by either wide local excision (WLE) with margins or Mohs micrographic surgery (MMS) [2,7]. This report highlights a case of DFSP diagnosed in the primary care setting, reviews the presentation, pathogenesis, diagnosis, and treatment of DFSP, compares DFSP to common conditions in its differential diagnosis, and reviews the literature on misdiagnosed and mismanaged cases of DFSP and their outcomes. 

## Case presentation

A 73-year-old woman, Fitzpatrick skin phototype V, presented to the primary care clinic with a three-year history of an asymptomatic, slowly enlarging nodular lesion located on the left upper thigh. At first, the lesion resembled a dime-sized pimple, but within the past year, the lesion progressed in size. The patient reported the growing lesion had become bothersome due to its size; however, it remained painless and nonpruritic without bleeding or drainage.

**Figure 1:**
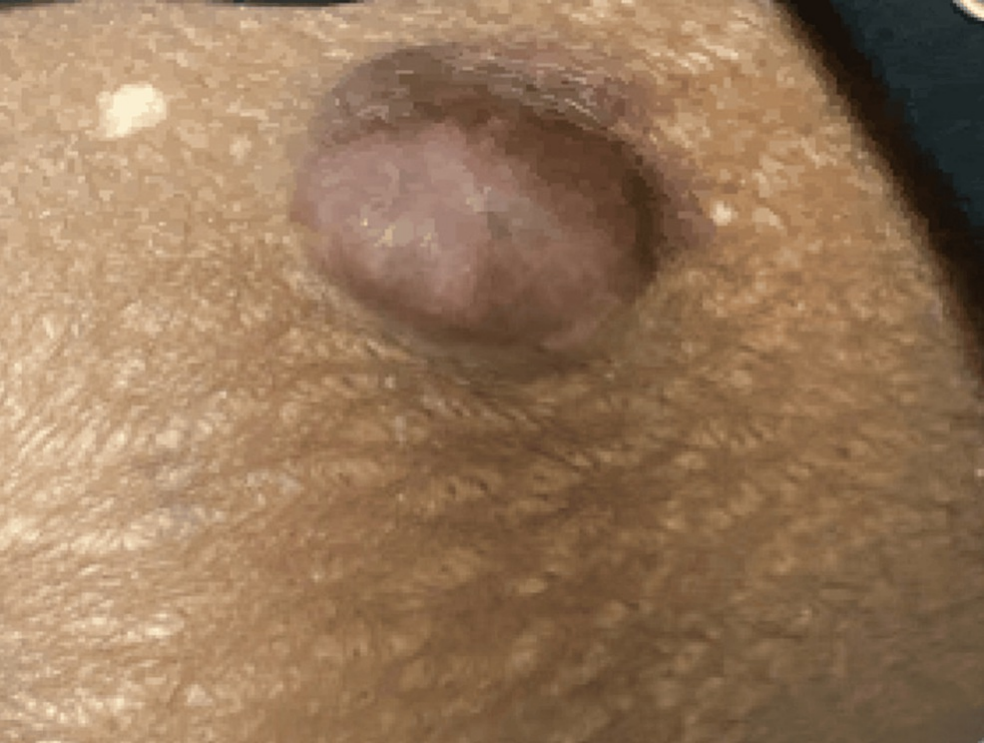
Physical exam showing a skin-colored to violaceous/hyperpigmented nodule on the superior anteromedial portion of the left thigh

**Figure 2:**
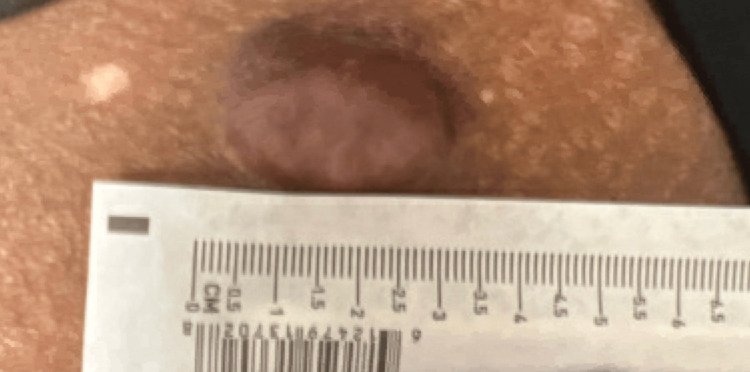
Measurement of lesion seen on physical exam

**Figure 3:**
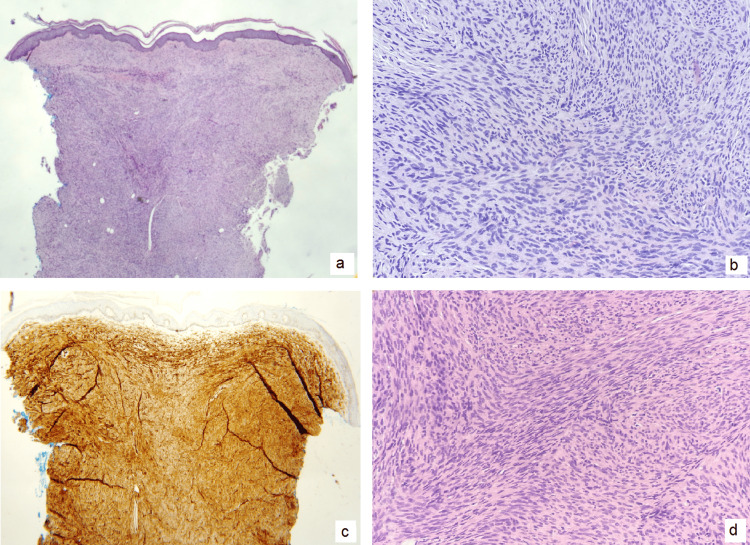
Microscopic images from left upper thigh punch biopsy demonstrating DFSP. (a) H&E stained section (x20 magnification) showing a dense dermal spindle cell proliferation and unremarkable overlying skin; (b, d) H&E stained sections (x200 magnification) showing a monomorphic spindle cell population with characteristic fascicular, storiform, and herringbone growth patterns. (c) Immunostaining for CD34 (40x magnification) showing diffuse immunoreactivity, compatible with diagnosis of DFSP. Lesion was not immunoreactive to S100, desmin, D2-40 (podoplanin), or EMA. H&E: hematoxylin and eosin; DFSP: dermatofibrosarcoma protuberans; EMA:epithelial membrane antigen

Physical examination showed a solitary, skin-colored to violaceous/hyperpigmented nodule on the superior anteromedial portion of the left thigh measuring 3.5 x 3.0 cm (Figure 1 and Figure 2). On palpation, the lesion was firm and nontender. The skin surrounding the lesion revealed hypopigmented striae and two small, depigmented macules at the 11 o’clock region. Patient consent for biopsy was obtained. A 4 mm punch biopsy was performed and sent to histopathology, which showed spindle cell proliferation (Figure 3) compatible with dermatofibrosarcoma protuberans. Immunohistochemical staining of the tissue sample was negative for desmin, epithelial membrane antigen (EMA), neuron-specific enolase (NSE), S100, and D2-40 and diffusely positive for CD34, confirming the diagnosis (Figure 3).

The patient was informed and educated on her diagnosis and referred to a dermatological surgeon for complete surgical excision. The patient was evaluated by Dermatology and agreed to proceed with WLE. The tumor was successfully removed and there has been no recurrence to date. She will continue to be followed closely by dermatology and primary care for signs of recurrence. 

## Discussion

Non-specialist practitioners must pay careful attention when diagnosing and treating skin lesions. Practitioners should be weary of skin lesions that are growing or changing, nonhealing, or inconsistent with a suspected clinical picture and evaluate such lesions further with biopsy. DFSP is a rare cutaneous soft tissue tumor that has a broad differential and may be inaccurately diagnosed and treated outside the dermatology setting.

Previous studies have demonstrated that greater than 90% of DSFPs arise in the setting of supernumerary ring chromosomes derived from chromosomes 17 and 22 or chromosome 17 and 22 rearrangements: t(17; 22) (q22; q13). This resulting chromosomal translocation where the collagen type Iα1 gene (*COL1A1*) fuses to the PDGFB leads to continuous activation of the PDGFRβ protein tyrosine kinase which drives DFSP cell growth [2,6]. However, additional genes (*CSPG2, PTK2B, COL1A2, COL6A3, PDGFD, EMILIN2, P53*, and *MDM2*) and signaling pathways (RAS-MARK and PI3k-Akt-mTOR) have been identified in the pathogenesis of DFSP. 

DFSP is uncommon and accounts for 0.1% of all malignancies and less than 2% of all sarcomas [2,4]. It frequently presents in young to middle-aged adults, aged 25-45, but has been reported in all age groups from infancy to the elderly [2]. Clinically, DFSP presents as an indolent, small, firm, skin-colored dermal plaque, subcutaneous thickening, or atrophic, non-protuberant lesion. Over several months to many years, the early lesions slowly enlarge to form protuberant, indurated, reddish-blue to violaceous nodules. DFSPs range in size from 0.5 cm to > 10 cm and typically present on the trunk (40-50%), followed by proximal extremities (30-40%) and head and neck (10-15%) [2-4,6]. 

Due to clinical similarities and overlaps in immunohistochemistry, including CD34 positivity, DFSPs must be differentiated from other benign and malignant lesions including, but not limited to, dermatofibromas, schwannomas, cutaneous neurofibromas, and solitary fibrous tumors (Table 1) [2]. Table 1 reviews lesions that present similarly to DFSP which may be included in its differential diagnosis. 

**Table 1:** Differential diagnosis of dermatofibrosarcoma protuberans αSMA: alpha-smooth muscle actin; STAT6: signal transducer and activator of transcription 6

Lesion	Clinical Features	Histology	Immunohistochemistry
Dermatofibroma	Firm, non-tender, tan-pink/reddish-brown skin lesion with a smooth surface. Usually less than or equal to 1 cm. Classically presents with “dimple” sign. Patients often report local trauma at site of lesion [2].	Localized proliferation of spindle-like fibrous cells surrounded by histiocytoid cells within the dermis. Distinguishing characteristic is the presence of trapped collagen bundles (“collagen balls”) within and between fascicles of spindled fibrous cells [2,8].	XIIIa(+) CD34(+) [2]
Schwannoma	Round, ovoid, well-circumscribed solitary tumors. Frequently on the extremities. May be associated with neurofibromatosis 2 (NF2) [2].	Well-circumscribed with areas composed of fascicles of Schwann cells with spindle morphology, surrounded by a capsule. Areas of nuclear alignment or palisading. May display Verocay bodies [2,9].	S100(+) [2]
Cutaneous Neurofibroma	Skin-colored, painless, slow-growing, soft, rubbery nodule [2].	Multiple cell types including Schwann cells, perineural-like cells, fibroblastic cells, and chopped axons interspersed with shredded carrot collagen, mast cells, and lymphocytes [2].	S100(+), CD34(+), Sox10(+), Collagen IV(+), αSMA(-), KIIIa(-) [2]
Solitary Fibrous Tumor	Painless, slow-growing mass. May cause nerve compression [2].	Haphazardly arranged spindle or oval-shaped cells lying in a variably collagenous stroma in a pattern-less arrangement OR Spindle or oval-shaped cells interspersed in a network of branching and hyalinized staghorn small vessels [2,10]	CD34(+), CD99(+), STAT6(+). Vimentin (+) S100 (-), Desmin (-) [2].

Standard treatment for DFSP involves surgical resection by either WLE with margins or MMS [2,4]. In certain clinical cases and non-resectable tumors, targeted therapy with imatinib and radiation has also been used [2,7]. While WLE was previously the standard of care, it was associated with high recurrence rates [4]. Mohs surgery, which leads to smaller surgical defects and less complicated reconstructions, is the preferred treatment option for DFSP to date. A study by Serra-Guillen et al. investigated 222 cases of DFSP treated with MMS and found that the average number of stages needed for tumor clearance was 1.47 with most cases (63.5%) requiring a single stage [3]. 

Multiple studies show lower recurrence rates and smaller, less complex surgical defects with MMS compared to WLE. Data from the Spanish Registry of Mohs Surgery (REGESMOHS) revealed an overall recurrence rate of 0.97 cases/100 person-years (95%CI 0.36-2.57). Of note, recurrence rates of lesions treated with slow MMS were lower and frozen MMS were higher, 0.74 (95%CI 0.18-2.95) cases/100 person-year vs 1.56 (95%CI 0.39-6.24) cases/100 person-year, respectively. Additionally, the study found that recurrent tumors demonstrated deeper invasion into the subcutaneous tissue or fascia and required a higher number of Mohs surgery stages, more than two in 75% of cases [4]. Other studies have found similar results which further supports MMS as the most appropriate treatment for DFSP. 

Although DFSP generally has a good prognosis, delayed diagnosis and treatment can lead to local invasion of other tissues (fascia, muscle, periosteum, bone) and more rarely, metastasis to other organs [2]. Patients with DFSP often receive inaccurate and delayed diagnoses due to the lesions’ often deceptively benign appearance, leading to improper management and inappropriately delayed treatment of these rare soft-tissue tumors [11].

Misdiagnosed and/or improperly managed cases of DFSP and their clinical outcomes. 

According to a study by David et al., the median delay in diagnosis of DFSP is three to five years, with lesions initially presenting as non-protuberant and experiencing longer intervals until diagnosis [11]. The study included data from 214 individuals who were part of a DFSP Facebook Support Group (FBSG); participants completed a multiple-choice survey investigating time from the appearance of the lesions to their diagnosis, surgical scar size, incidences of metastasis, incidences of recurrence, and other associated symptoms. Most participants (n=167; 78.0%) first noticed the lesions themselves, with the remainder of respondents having their lesions noticed by their clinician (2.3%), imaging (0.9%), or by a family member, friend, or other person (18.75%). In this study, “clinician” referred to physicians, nurse practitioners, and physician assistants. Median time from first noticing the tumor to seeking evaluation by a clinician was one year, with a range of 1-31 years. Median total time from first noticing the lesion to receiving a diagnosis was four years, with a range of < 1-42 years [11]. 

Most respondents (52.3%) in their study believed they were misdiagnosed at some point, with 95.5% of those individuals believing they were misdiagnosed prior to biopsy. Potential misdiagnoses came from dermatologists (32.7%), primary care clinicians (74.8%), and other non-specified clinicians (25.2%). Misdiagnosis by more than one type of clinician was possible. The most common pre-biopsy diagnoses given to respondents included but were not limited to cyst, lipoma, scar, dermatofibroma, and keloid. Respondents reported multiple office visits (five or more in 19.6%) prior to receiving a biopsy of their lesion as well as visiting up to five different clinicians before biopsy (8.9%) [11]. This data further necessitates the importance of educating non-specialist clinicians on DFSP, including its possible presentations and proper management. 

A 2020 case out of Bejing, China, by Lin et al., documented a case of DFSP misdiagnosed for 10 years as post-inflammatory hyperpigmentation [12]. The patient was a 33-year-old female with a 1.6 x 1.3 cm erythematous-to-bluish, ill-defined, depressed plaque on the left upper back. A biopsy of the lesion was performed which revealed histopathologic and immunohistochemical findings consistent with DFSP. Of noteworthy importance in this case, biopsy results showed reduced dermal thickness and dendritic cells with abundant melanin which were S-100 positive. These unique characteristics established a diagnosis of atrophic-pigmented DFSP. Atrophic DFSP is characterized as a slow-growing depressed plaque with > 50% decrease in dermal thickness compared to the surrounding tissue. Pigmented DFSP, also known as Bednar tumor, presents as a bluish plaque with melanin-laden dendritic cells on histology. At the time of diagnosis, only seven cases of atrophic-pigmented DFSP had been reported on PubMed. This case highlights the importance of performing a biopsy of any solitary, hyperpigmented lesion that does not resolve spontaneously, even if malignancy is not clinically expected. 

A 2022 report by Zhang et al. describes a case of DFSP in a 10-year-old boy misdiagnosed as a sebaceous adenoma and managed surgically by routine resection [13]. The left upper abdominal wall lesion was found accidentally and was asymptomatic until the lesion began enlarging and the patient began experiencing fevers and itching. An ultrasound of the mass showed a hypoechoic mass in the subcutaneous left upper abdomen. Color Doppler revealed blood flow within and around the mass. The mass was considered to be a sebaceous adenoma and was excised by local resection. Histopathologic examination was consistent with DFSP and the patient was readmitted to the hospital for extended resection of the lesion, at which time negative margins were obtained. Although DFSP is rarer in children, it should still be considered when diagnosing growing skin lesions in children. This case suggests that the presence of blood flow in a clinically benign-appearing lesion should warrant wide excision to avoid multiple procedures and hospitalizations in children. 

Another case in 2016, by Kimura et al., reports a 46-year-old male with DFSP misdiagnosed as a keloid [14]. The patient underwent several intralesional steroid injections for a prolonged period with minimal improvement. These lesions were subsequently biopsied and showed typical pathologic features of DFSP including cells in a storiform pattern with infiltration or into the subcutis and CD34 positivity, and proliferating cells in a storiform pattern with low cellularity, respectively. This patient’s lesion showed unique clinical features highlighting that DFSP is not always protuberant. Lesions with indurated erythema, atrophic plaques, and keloid-like nodules may represent less common forms of DFSP and should be biopsied to prevent misdiagnosis and delay of treatment.

In a case from Turkey in 2018, a 19-year-old female patient with no known history of trauma or scarring in the region presented with a one-year history of a “small red pimple” on the chest which was clinically diagnosed as a keloid and excised by general surgery [15]. Surgical pathology revealed DFSP. At that time, a CT scan was done for staging and showed no distant metastasis. After a period of time, she was reevaluated for possible recurrence. The lesion was completely resected and there was no evidence of recurrence at the six-month mark. DFSP can oftentimes be mistaken for keloid, particularly if it grows to reach 50 mm in size [15]. It is important for providers to perform a thorough examination, including skin biopsy, for the accurate diagnosis of skin conditions. It is also critical that lesions with histology consistent with DFSP be completely resected with MMS or WLE to minimize recurrence and the need for subsequent excisions.

## Conclusions

DFSP is a rare soft tissue tumor that can affect individuals in all age groups. Practitioners, especially non-specialist providers, should consider DFSP when evaluating lesions resembling DFSP, including atrophic and pigmented types, with no history of trauma or scar. Delayed diagnosis may lead to local destruction of tissue and more rarely, distant metastasis. Tumors diagnosed as DFSP should be properly treated with MMS or wide local excision, although MMS has lower recurrence rates and better cosmetic outcomes. Patients should be followed closely after excision due to DFSP’s increased tendency for recurrence.
